# The Sheaths of *Methanospirillum* Are Made of a New Type of Amyloid Protein

**DOI:** 10.3389/fmicb.2018.02729

**Published:** 2018-11-13

**Authors:** Line Friis Bakmann Christensen, Lonnie Maria Hansen, Kai Finster, Gunna Christiansen, Per Halkjær Nielsen, Daniel Erik Otzen, Morten Simonsen Dueholm

**Affiliations:** ^1^Interdisciplinary Nanoscience Center (iNANO), Department of Molecular Biology and Genetics, Aarhus University, Aarhus, Denmark; ^2^Center for Microbial Communities, Department of Chemistry and Bioscience, Aalborg University, Aalborg, Denmark; ^3^Section for Microbiology, Department of Bioscience, Aarhus University, Aarhus, Denmark; ^4^Section for Medical Microbiology and Immunology, Department of Biomedicine, Aarhus University, Aarhus, Denmark

**Keywords:** archaea, methanogen, functional amyloid, sheath protein, homologs

## Abstract

The genera *Methanospirillum* and *Methanosaeta* contain species of anaerobic archaea that grow and divide within proteinaceous tubular sheaths that protect them from environmental stressors. The sheaths of *Methanosaeta thermophila* PT are composed of the 60.9 kDa major sheath protein MspA. In this study we show that sheaths purified from *Methanospirillum hungatei* JF-1 are regularly striated tubular structures with amyloid-like properties similar to those of *M. thermophila* PT. Depolymerizing the sheaths from *M. hungatei* JF-1 allowed us to identify a 40.6 kDa protein (WP_011449234.1) that shares 23% sequence similarity to MspA from *M. thermophila* PT (ABK14853.1), indicating that they might be distant homologs. The genome of *M. hungatei* JF-1 encodes six homologs of the identified MspA protein. Several homologs also exist in the related strains *Methanospirillum stamsii* Pt1 (7 homologs, 28–66% sequence identity), *M. lacunae* Ki8-1 C (15 homologs, 29–60% sequence identity) and *Methanolinea tarda* NOBI-1 (2 homologs, 31% sequence identity). The MspA protein discovered here could accordingly represent a more widely found sheath protein than the MspA from *M. thermophila* PT, which currently has no homologs in the NCBI Reference Sequence database (RefSeq).

## Introduction

Archaea differ from both Bacteria and Eukarya because of their unique cell properties. They lack Eukarya’s membrane-enclosed nucleus, and unlike bacterial cell walls, they rarely contain peptidoglycans (Kandler and Konig, 1978) and their phospholipid membranes are made up of ether-linked phytanols (Makula and Singer, 1978). Even though archaea were initially identified in extreme environments such as hot springs and salt brines, they have now been identified in practically all habitats (Cavicchioli, 2011). Most archaea have a so-called surface layer (S-layer) cell wall structure made up of a single protein or glycoprotein with a molecular mass in the 40–200 kDa range (Albers and Meyer, 2011). The cell envelope of archaea can be composed of these S-layers alone or they can be combined with polysaccharides like pseudomurein as seen for *Methanothermus fervidus* (Stetter et al., 1981).

Even more complex than regular S-layer cell walls are the proteinaceous sheaths found in some methanogenic archaea. *Methanospirillum* and *Methanosaeta* are evolutionarily distant anaerobic archaea, which share the ability to produce methane. They can grow and divide within proteinaceous, tubular sheaths that enclose an entire chain of cells (Zeikus and Bowen, 1975; Beveridge et al., 1985, 1986). Individual cells have single-layer cell walls and are separated by spacer plugs within the sheaths (Beveridge and Graham, 1991). The morphology of purified sheaths is similar for both *Methanospirillum* and *Methanosaeta*, showing hollow tubes composed of individual rings that are responsible for their hoop-like appearance (Beveridge et al., 1985, 1986; Sprott et al., 1986). The sheaths tend to fracture longitudinally between individual hoops (Sprott et al., 1986; Southam and Beveridge, 1991, 1992), suggesting that the intermolecular connections between hoops are the weakest part of a sheath. Despite significant morphological similarities there are also clear differences. Amino acid composition analysis of sheaths from *Methanosaeta concilii* and *Methanospirillum hungatei* has revealed that they differ in the ratio of acidic/basic aa as higher proportions of alanine and aspartate and lower proportions of glutamate, histidine and arginine are seen for *M. hungatei* (Patel et al., 1986). In addition, the spacer plugs are different in term of subunit arrangement and stability toward NaOH. *M. hungatei* spacer plugs have a complex hexagonal symmetry while those from *M. concilii* resemble concentric rings (Patel et al., 1986). To release the spacer plugs from *Methanospirillum* sheaths, mild alkali treatment (0.1 M NaOH) is sufficient while plugs from *Methanosaeta* sheaths are not even released by treatment with 5 M NaOH (Patel et al., 1986).

It has long been recognized that the protein sheaths are extremely stable and resist dissolution by denaturing conditions such as 6 M urea, 6 M guanidinium chloride, and 10 M lithium thiocyanate hydrate (LiSCN) as well as they also resist proteases and other enzymes (Beveridge et al., 1985). The elastic properties of the sheaths from *M. hungatei* lead to very large Young’s modulus values between 2 × 10^10^ and 4 × 10^10^ N/m^2^ (corresponding to between 20 and 40 GPa) (Xu et al., 1996). This is in the same range as some amino acid molecular crystals (Azuri et al., 2015) and hemp fibers (Liu et al., 2015). This is partly due to the presence of disulfide bonds that keep sheath rings/hoops together and therefore require strong reducing conditions to be split (Sprott et al., 1986; Southam and Beveridge, 1991; Dueholm et al., 2015).

Another reason for the great stability of the sheaths could be that they are composed of functional amyloids, which often require concentrated formic acid (Collinson et al., 1991; Chapman et al., 2002), trifluoroacetic acid (Claessen et al., 2003; Jordal et al., 2009) or hexafluoroisopropanol (HFIP) (Cegelski et al., 2009) to depolymerize. This was shown to be the case for the major sheath protein MspA (ABK14853.1) from *M. thermophila* PT (Dueholm et al., 2015). Sheaths of this archaeon were separated and depolymerized with DTT and 90–100% formic acid, and MspA was subsequently identified by mass spectrometry (Dueholm et al., 2015). Like other functional amyloids, recombinant MspA extremely easily aggregates and can fibrillate under a wide range of conditions (pH 3–9, low/high ionic strength). The recombinant protein fibrillates within 1 min when diluted from concentrated guanidinium chloride, producing aggregates with features characteristic of amyloid structures (Maji et al., 2009; Moran and Zanni, 2014), such as fibrillar morphology in TEM images and secondary structure dominated by β-sheets with a major peak around 1625 cm^−1^ in FTIR spectroscopy.

Functional amyloids are found in up to 40% of all bacterial biofilm (Larsen et al., 2007) where they e.g., increase mechanical stiffness and hydrophobicity (Zeng et al., 2015). Polymerization of amyloid proteins requires no energy input, making it metabolically attractive. However, the methanogens also need to take up metabolic substrates (e.g., H_2_ and CO_2_) and release waste products like CH_4_. This has been suggested by Dueholm et al. (2015) to happen through the more flexible regions of the sheaths where individual hoops are connected by disulfide bonds (Schauer et al., 1982). A different study suggested that nutrient and waste products were released by stretching/expansion of the sheaths when the cells reach a certain pressure (Xu et al., 1996). The true mechanism might be a combination of the two models.

Because of the great similarities between *Methanosaeta* and *Methanospirillum* sheath structures, we wanted to investigate the *M. hungatei* JF-1 sheath in more detail. *M. hungatei* JF-1 sheaths can be disassembled using a reducing agent like DTT and the strong base NaOH (Beveridge et al., 1985; Sprott et al., 1986). Using this approach, we identify a previously uncharacterized 40.6 kDa protein, which we propose to be a novel MspA protein. The new MspA appears to be highly amyloidogenic, in correlation with the functional amyloid nature of MspA from *M. thermiphila* PT (Dueholm et al., 2015). Importantly, we also identified 24 homologs in closely related methanogenic strains, indicating that this sheath-forming MspA is more widely used than the *M. thermophila* PT MspA, which has no obvious homologs in the current version of the RefSeq database (Dueholm et al., 2015).

## Materials and Methods

### Growth of *Methanospirillum hungatei* JF-1

A pure culture of *M. hungatei* JF-1 (DSM-864) was obtained from the German Collection of Microorganisms and Cell Cultures (DSMZ) and cultivated in a defined pre-reduced synthetic acetate (SA) broth supplemented with 80% H_2_ and 20% CO_2_ twice a week (Patel et al., 1976) at 37°C under anaerobic conditions. Growth was regularly checked using a phase contrast microscope.

### Purification of Sheaths

The pellet from 200 mL of pure culture was resuspended in 1 mL phosphate-buffered saline (PBS) (10 mM PO_4_^3-^, 137 mM NaCl, 2.7 mM KCl, and pH 7.4), ultracentrifuged (21,000 ×*g*, 20 min) and the supernatant was discarded. The pellet was resuspended in 1 mL enzyme mix (0.1 mg/mL RNase, 0.1 mg/mL DNase, 1 mM MgCl_2_ and 0.1% Triton X-100 in 10 mM Tris–HCl, and pH 8.0) and cell lysis was performed with three cycles of freeze-thawing using a −80°C freezer and a 37°C water bath. The cell solution was kept at each condition for 30 min. followed by incubation at 37°C for 2 h to allow enzymatic degradation. Sodium dodecyl-sulfate (SDS) was added to 2% and the solution was boiled for 5 min at 95°C to dissolve non-amyloid proteins before centrifugation (30,000 rpm, 20 min, 20°C). The pellet was resuspended in 10 mM Tris–HCl, pH 8.0 and again subjected to 2% SDS, boiled and centrifuged. Then the solution was washed three times by resuspending the pellet in buffer (10 mM Tris–HCl, pH 8.0) and centrifuging it (30,000 rpm, 20 min, 4°C). After the last wash, the pellet was resuspended in 100 μL buffer containing 1 mg/mL sodium azide and investigated with TEM and FTIR spectroscopy.

### Transmission Electron Microscopy (TEM)

Five microliter sample was applied to a 400-mesh carbon-coated glow-discharged Ni grid. Grids were stained after 30 s with 1% phosphotungstic acid (pH 7.0) and blotted dry on filter paper. Samples were viewed in a transmission electron microscope (JEM-1010, JEOL) operating at 60 kV. Images were obtained using an Olympus KeenViewG2 camera.

### Fourier-Transform Infrared (FTIR) Spectroscopy

Two microliter of sample was dried onto an Attenuated Total Reflection crystal with dry nitrogen on a Tensor 27 FTIR instrument (Bruker Optics, Billerica, MA, United States). OPUS version 5.5 was used to process the data, including calculating atmospheric compensation, baseline subtraction, and second-derivative analysis. All spectra were made as accumulations of 68 scans with a resolution of 2 cm^−1^ in the range of 1,000–3,998 cm^−1^. Only the amide I band (1,600–1,700 cm^−1^ range, comprising information about secondary structure) is shown.

### Binding of the Amyloid-Specific Antibody WO1

The amyloid-specific conformational antibody WO1 was used to confirm the presence of amyloid epitopes on intact *M. hungatei* JF-1 cells. The binding assay was performed as previously described (Larsen et al., 2008). In short, cells were pelleted by centrifugation (21,000 ×*g*, 30 min) and washed in PBS before resuspension in a PBS solution containing 1% (w/v) gelatin. 10 nM WO1 and 0.05% (w/v) Tween20 were added to the sample after a 1 h incubation at 37°C and incubated for an additional 2 h at 37°C. Primary antibody was removed by three washing steps using a PBS solution containing 1% (w/v) gelatin and 0.1% (v/v) Triton X-100. The secondary antibody used was the Alexa Fluor^®^ 488 goat anti-mouse immunoglobulin M (IgM) (μ chain) from Molecular Probes (1:256 dilution) with which the samples was incubated for 1 h at 37°C together with 0.025% Tween20 and 1% (w/v) gelatin. Samples were washed three times before analysis on a confocal laser scanning microscope (LSM) (510-META) (Carl Zeiss) with an argon ion laser of 458 and 488 nm. To calibrate the microscope settings, a sample of activated sludge sample, containing both amyloid-positive and amyloid-negative cells, was used as previously described (Larsen et al., 2008). To check for unspecific binding of secondary antibody to the *M. hungatei* JF-1 sheaths, samples were also incubated without the primary antibody.

### Disassembly of Sheaths and SDS–PAGE

The sheaths from *M. hungatei* were disassembled using NaOH as previously described (Beveridge et al., 1985). Briefly, purified sheaths were mixed with 66 mM DTT (pH 8.0) and incubated at 37°C for 30 min. After reaching room temperature, 1 M NaOH was added and the samples were incubated for additionally 10 min at 22°C before neutralizing with HCl. The samples were lyophilized and dissolved in a special reducing SDS–PAGE loading buffer containing 8 M urea (75 mM Tris, 0.6% (w/w) SDS, 15% (v/v) glycerol, 7.5% (v/v) β-mercaptoethanol, 0.9 mg/L bromphenol blue, 8 M urea, and pH 6.8) as previously described (Dueholm et al., 2015). Samples were loaded on an AnyKD gel (Bio-Rad) and run for 40 min at 140 V before staining with Coomassie Brilliant Blue G250.

### Mass Spectrometry

Mass spectrometric analysis of the sheath proteins was performed as previously described (Dueholm et al., 2015). Briefly, all bands were cut from the gel into 1 × 1 mm pieces and washed with 50 mM NH_4_HCO_3_ and 30% (v/v) acetonitrile. Reduction and alkylation were performed with DTT and iodoacetamide, respectively, before drying the gel pieces. Rehydration was done in a 10 ng/μL sequencing grade trypsin solution (Promega) and trypsin digestion was performed overnight at 37°C. The resulting peptides were extracted, combined and dried before reconstitution in a liquid chromatography-mass spectrometry (LC-MS) sample buffer (2% acetonitrile, 0.1% formic acid). A Dionex Ultimate 3,000 nanoLC system (Thermo Scientific) connected to a Quadropole Orbitrap (Q Exactive) mass spectrometer with a NanoSpray Flex ion source (Thermo Scientific) was used to analyze the extracted peptides. Peptides were trapped on an Acclaim PepMap100 C 18.5 μm column and separated on a 50 cm Acclaim PepmapRSLC 17-μm column (Thermo Scientific) before being subjected to nano-electrospray with a Picotip Silicatip emitter (New Objectives). Data acquisition and protein identification were performed as previously described (Dueholm et al., 2015) but in this case using the Mascot server v2.3 (Matrix Sciences) to search against the Uniprot database with the complete genome for *M. hungatei* JF-1 (METHJ, released on February 18th, 2006). The Exponentially modified Protein Abundance Index (EmPAI) (Ishihama et al., 2005) was used to quantitate the proteins identified by MS/MS.

### Genome Sequencing of Other *Methanospirillum* Type Strains

DNA from *M. lacunae* Ki8-1 (DSM-22751) and *M. stamsii* Pt1 (DSM-26304) were obtained from DSMZ. Paired-end libraries were prepared using the Nextera DNA Library Prep Kit (Illumina), and sequencing was performed using a MiSeq sequencer (Illumina, Germany). The reads were trimmed for adapters and quality and *de novo* assembled using the built-in tool of CLC Genomics Workbench v.9.5.5 (QIAGEN, United States) using the default settings. The average coverage of the assemblies was 150× and 42× for *M. lacunae* Ki8-1 and *M. stamsii* Pt1, respectively. Annotation was done using the NCBI prokaryotic genome automatic annotation pipeline (12). The Whole Genome Shotgun projects have been deposited at DDBJ/EMBL/GenBank under the accession no. QGMY00000000 and QGMZ00000000 for *M. lacunae* Ki8-1 and *M. stamsii* Pt1, respectively. The version described in this paper is the first version, QGMY01000000 and QGMZ01000000.

### Bioinformatic Analysis of Identified Sheath Proteins

Sheath protein homologs in the RefSeq Database version 2.8.0 and in the genomes of the two *Methanospirillum* type strains sequenced in this study were identified using the blastp algorithm (Altschul et al., 1990). Protein alignments were carried out using the CLC Main Workbench 6.0 (CLC bio). The gap opening cost was set to 10 and the gap extension cost to 1. The RADAR (Rapid Automatic Detection and Alignment of Repeats) tool (Andreas and Liisa, 2000) was used to identify protein imperfect repeats. AmylPred2 (Tsolis et al., 2013) and AmyloGram (Burdukiewicz et al., 2017) were used to evaluate the amyloidogenicity of sheath proteins. Signal peptide prediction was performed with SignalP 5.0 (not yet available online). SignalP 5.0 is an extension of the existing SignalP 4.1^1^ (Nielsen, 2017) and offers the ability to search specifically against the archaea organism group.

## Results

### Intact *Methanospirillum hungatei* JF-1 Bind the Amyloid-Specific Antibody WO1

**FIGURE 1:**
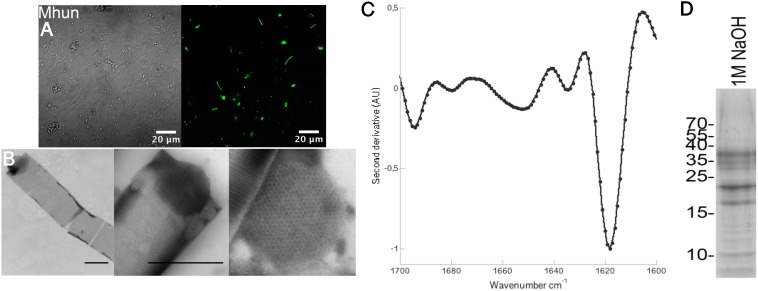
*Methanospirillum hungatei* JF-1 sheaths are composed of functional amyloids that require DTT and NaOH for dissolution. **(A)** Binding of the amyloid-specific antibody WO1 to intact *M. hungatei* JF-1 (Mhun) filaments (right). The left side is a differential interference contrast image. **(B)** TEM images of purified sheaths from *M. hungatei* JF-1 at 8,000X (left) and 25,000X magnification (middle). A zoom in on a spacer plug with its hexagonal arrangement of subunits is shown on the right. Scale bars correspond to 500 nm. **(C)** Normalized second derivative FTIR spectra of purified sheaths. **(D)** Gel bands after depolymerization of purified sheaths using DTT and NaOH.

Because of the wide abundance of functional amyloids in bacterial biofilms (Larsen et al., 2007), several different antibodies have been developed that recognize the amyloid fold (O’Nuallain and Wetzel, 2002; Kayed et al., 2007; Miles et al., 2008). One of these, the WO1 antibody, has previously been shown to bind to the methanogen *M. thermophila* PT (Dueholm et al., 2015). Binding of the WO1 antibody to the intact *M. hungatei* JF-1 gave a clear green fluorescent signal, indicating that this methanogen is also enclosed within a functional amyloid protective layer (Figure 1A). No unspecific binding to the secondary antibody was observed (Supplementary Figure S2).

### Purified Sheaths From *M. hungatei* JF-1 Are Tubular Structures Composed of 11–17 nm Wide Hoops

The protocol used to purify the *M. hungatei* JF-1 sheaths was originally used for purification of functional amyloids in *Pseudomonas* (Fap fibrils) (Dueholm et al., 2010) and has more recently been used to purify the sheaths of *M. thermophila* PT (Dueholm et al., 2015). It takes advantage of the high stability of the sheaths (and of functional amyloids in general), enabling them to withstand boiling in 2% SDS – a treatment that dissolves other non-amyloid protein structures. As revealed by TEM, the sheaths are tubular structures of highly regular striation (Figure 1B). The striations show 11–17 nm spacings as have previously been observed for *M. hungatei* GP1 (Beveridge et al., 1985; Southam and Beveridge, 1991), *M. thermophila PT* (Dueholm et al., 2015), and *M. concilii* (Patel et al., 1986). We have previously proposed these striations to consist of individual hoops or rings that stack together. The spacer plugs that separate individual cells within the sheaths could also be observed with TEM and clearly showed the hexagonal arrangement of the spacer plug subunits characteristic of *M. hungatei* (Patel et al., 1986; Figure 1B). FTIR spectra of the purified sheaths (Figure 1C) produced an intense peak at 1,618 cm^−1^ in the amide I region (between 1,600 and 1,700 cm^−1^) which is consistent with the characteristic amyloid cross-β structure. Conventional β-sheets normally absorb >1,630 cm^−1^, but the greater size and higher general order of amyloid cross-β sheets shifts the frequency range to lower wavenumbers (Moran and Zanni, 2014).

### *Methanospirillum hungatei* JF-1 Sheaths Are Composed of a 40.6 kDa Major Sheath Protein

**FIGURE 2:**
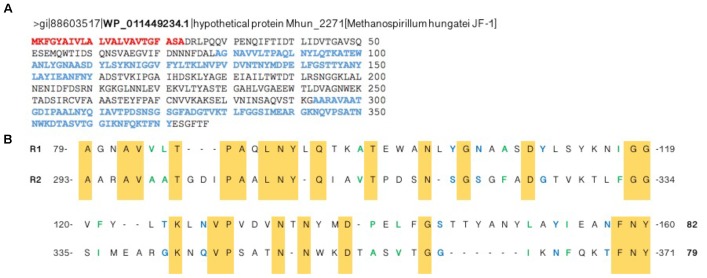
A single protein, MspA, with two imperfect repeats makes up the *Methanospirillum*. **(A)** Disassembly of the purified sheaths followed by SDS–PAGE and mass spectrometry analysis led to the identification of the hypothetical protein WP_011449234.1, now referred to as MspA. The signal peptide residues are colored red while the two imperfect repeats, identified with the online tool RADAR (Andreas and Liisa, 2000), are colored blue. **(B)** Alignment of the two imperfect repeats. Conserved residues are colored yellow while residues with similar properties are shown in green (small and/or hydrophobic) or blue (polar + Gly).

The sheaths of *M. hungatei* are extremely stable (Beveridge et al., 1985) but can be disassembled by combining the reducing agent DTT with 1 M NaOH (Figure 1D; Beveridge et al., 1985). Analysis of the depolymerized sheaths on SDS–PAGE revealed several protein bands. Mass spectrometry analysis revealed that all proteins of these bands were hydrolysis products that originated from a single 40.6 kDa (377 aa) protein, WP_011449234.1, that has not yet been characterized (Figure 2 and Supplementary Table S1). We name this protein Major sheath protein A (MspA) to be consistent with the *M. thermophila* PT nomenclature (Dueholm et al., 2015). Consistent with previous reports (Sprott et al., 1986; Southam and Beveridge, 1991), the sheaths require DTT to dissolve, but since each individual MspA monomer only contains one Cys residue at position 271 (C271), we deduce that the MspA proteins must form intermolecular disulfide bonds. This is in good agreement with our model for how MspA protein organize in the sheaths of *M. thermophila* PT where we propose that disulfide bonds link the hoops together (Dueholm et al., 2015). The two Cys residues of *M. thermophila* MspA are located in flexible, non-core regions of the fibrils. It remains to be determined if this is also the case for the C271 in *M. hungatei* JF-1 MspA. Alignment of the MspA protein sequences from *M. hungatei* JF-1 (WP_011449234.1) and *M. thermophila* PT (ABK14853.1) using the EMBOSS needle tool showed that the two proteins share 14% sequence identity and 23% sequence similarity, indicating that they might be distant homologs.

### MspA From *Methanospirillum hungatei* JF-1 Contains Imperfect Repeats

The open reading frame for MspA (Mhun_2271) is not part of an operon structure, unlike both the curli system in *Escherichia coli* and the Fap system in *Pseudomonas* (Hammar et al., 1995; Chapman et al., 2002; Dueholm et al., 2010, 2013). Yet MspA shares other features typical of known functional amyloid proteins. The functional amyloids CsgA (major subunit of curli), FapC (major subunit of Fap fibrils), chaplins (proteins involved in aerial hyphae formation in *Streptomyces coelicolor* A3) and HpaG (hairpin protein from *Xanthomonas axonopodis* pv. glycine) all have more or less conserved imperfect repeats in their primary sequence (Dueholm et al., 2013). Two imperfect repeats, R1 and R2, could also be identified in MspA between residues 79–160 and 293–371, respectively (Figure 2).

The MspA repeats are longer (≈80 aa) and less conserved than those seen for CsgA (19 aa), FapC (34 or 48 aa) and the chaplin proteins (41 aa) (Supplementary Figures S3, S4). The MspA repeats contain conserved G, A, N, and Q residues as seen in CsgA and FapC (Supplementary Figure S3), but also many small hydrophobic aa like V and P as seen for the chaplin proteins (Supplementary Figure S4).

### The *Methanospirillum hungatei* JF-1 MspA Sequence Has Regularly Spaced Amyloidogenic Regions

**FIGURE 3:**
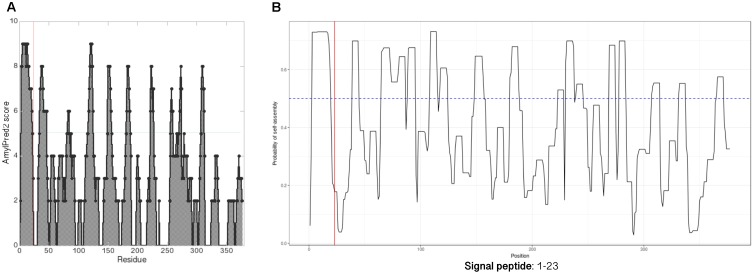
MspA has regularly spaced amyloidogenic regions. Analyzing the MspA (WP_011449234.1) sequence with **(A)** AmylPred2 (Tsolis et al., 2013) and **(B)** AmyloGram (Burdukiewicz et al., 2017) revealed several regions of predicted high amyloidogenicity (above the horizontal lines). The red, vertical lines indicate the signal peptide cleavage site position.

Amyloidogenic hotspots in proteins sequences can be predicted by bioinformatic tools like AmylPred2 (Tsolis et al., 2013) and AmyloGram (Burdukiewicz et al., 2017). Analysis with AmylPred2 showed that MspA has several amyloidogenic regions that are found with ≈25 aa spacings throughout the protein sequence (Figure 3A). As this corresponds roughly to the length of the repeats in CsgA there might be a structural resemblance between this archaeal amyloid and the curli fibrils (Supplementary Figure S2; Dueholm et al., 2012). The same trend with interspaced amyloidogenic regions was also observed with AmyloGram (Figure 3B). The first 23 aa of the N-terminal represent a signal peptide according to the SignalP 5.0 algorithm (Petersen et al., 2011; Supplementary Figure S1). Signal peptides often have high amyloidogenicity which can also be seen from both analyses (Figure 3).

### *M. hungatei* JF-1 Has Six Internal MspA Homologs

**FIGURE 4:**
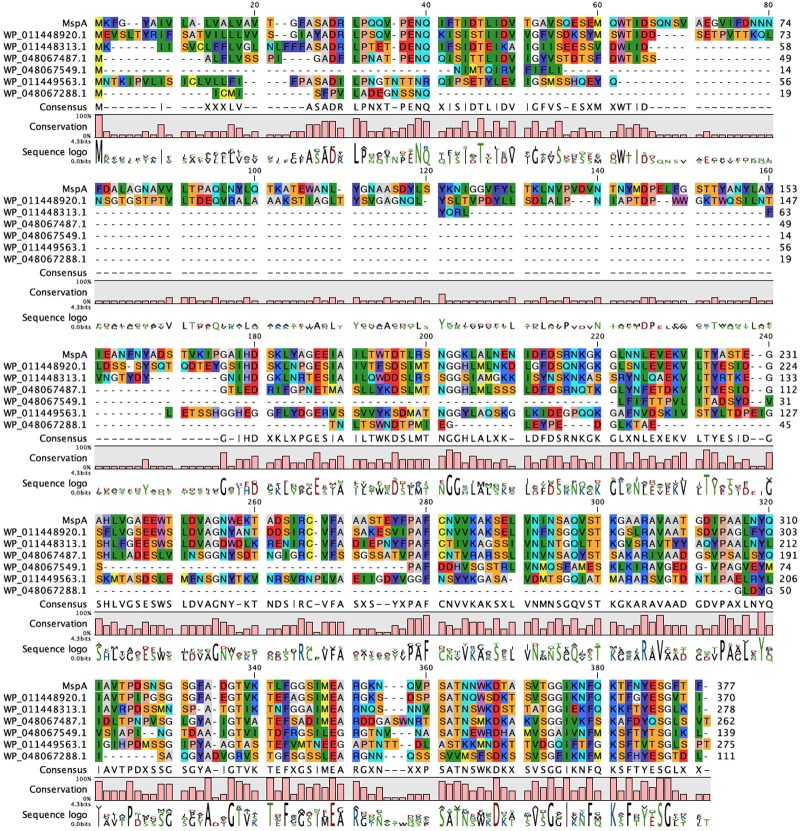
*Methanospirillum hungatei* have six internal MspA homologs. Sequence alignment of MspA from *M. hungatei* JF-1 (WP_011449234.1) and its internal homologs. Residues are colored according to Rasmol standards.

Both the curli system (CsgA-H) and the Fap system (FapA-F) have protein homologs within other classes of bacteria. The curli system is the most phylogenetically widespread, spanning at least four different phyla (Dueholm et al., 2012), while the Fap system has only been identified within the phylum Proteobacteria (Dueholm et al., 2013). Both CsgA and FapC display low sequence similarity, even between closely related species. The genome of *M. hungatei* JF-1 contains six homologs (30–56% sequence identity) of the novel MspA, referred to as internal homologs (Figure 4).

**FIGURE 5:**
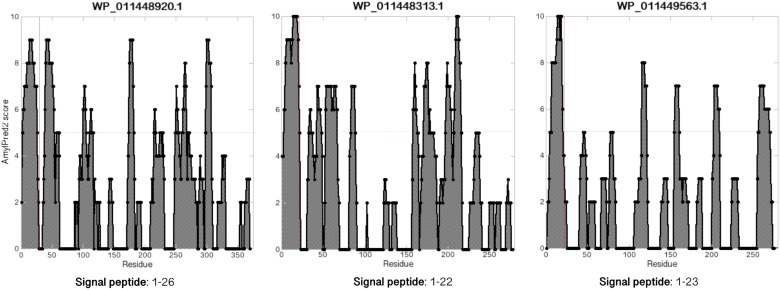
MspA internal homologs have regularly spaced amyloidogenic regions. Analyzing the MspA (WP_011449234.1) homologs found in *M. hungatei* with AmylPred2 (Tsolis et al., 2013) revealed several regions of predicted high amyloidogenicity (values above the horizontal blue lines). The red, vertical lines represent predicted signal peptide cleavage site positions.

These internal homologs vary between 111 and 370 aa in length. The three longest of the homologs (275–370 aa) all contain at least one Cys residue (two for WP_011448920.1 and three for WP_011448313.1) and were investigated with the SignalP and AmylPred2 tools to check for the presence of signal peptides and of amyloidogenic regions. All three proteins have a signal peptide region and have more or less regularly spaced amyloidogenic regions as seen for MspA (Figure 5).

Using the RADAR tool, in all five homologs (except the shortest) we identified imperfect repeats of either ∼20 aa (WP_011449563.1 and WP_048067549.1), ∼40 aa (WP_011448313.1 and WP_048067487.1), or 116 aa (WP_011448920.1) (Supplementary Figure S5). Repeats of ∼20 aa fits nicely with the repeat length of each of the five repeats in CsgA (Shewmaker et al., 2009; Evans and Chapman, 2014). Interestingly, even though the repeats of WP_011449563.1 and WP_048067549.1 are less conserved than the repeats in CsgA, they still contain some conserved G, S, and N residues (Supplementary Figure S5) and therefore may be able to fold up into a β-sheet-turn-β-sheet motif as seen for CsgA (Evans and Chapman, 2014). The longer repeats seen for the other homologs could contain multiples of the β-strand-turn-β-strand motifs.

### Closely Related *Methanospirillum* Strains Also Contain MspA Homologs

**FIGURE 6:**
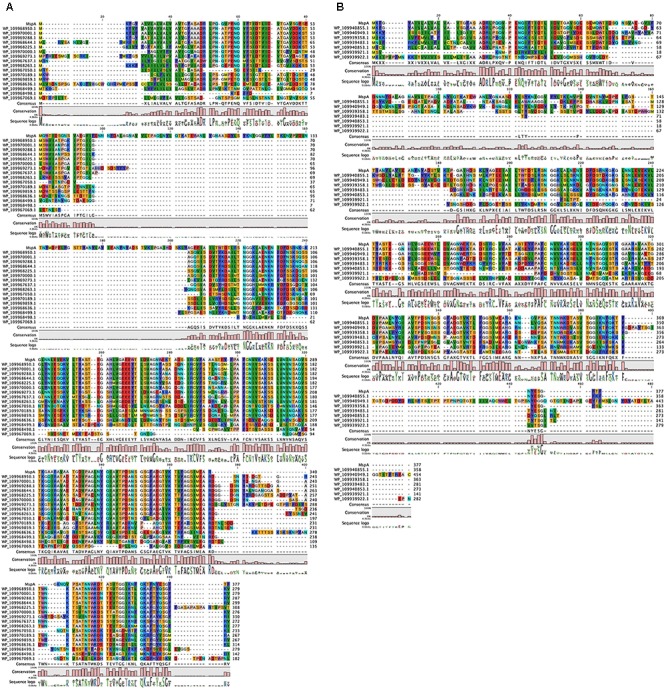
Other *Methanospirillum* strains encode homologs of the *M. hungatei* JF-1 MspA protein. Sequence alignment of MspA from *M. hungatei* JF-1 (WP_011449234.1) with **(A)** its 16 homologs in *M. lacunae* and **(B)** 7 homologs in *M. stamsii*. Residues are colored according to Clustal standards.

We next wanted to identify functional amyloid in closely related *M. lacunae* Ki8-1 and *M. stamsii* Pt1. Although we were able to cultivate these strains, their sheaths seemed to be even more stable than those of *M. hungatei*, since we were not able to depolymerize them using either the NaOH based method described in this study or by the formic acid treatment used for *M. thermophila* PT (Dueholm et al., 2015). We therefore decided to identify the sheath proteins in these strains using genome sequencing and bioinformatics. Accordingly, we sequenced the genomes of both strains. We were then able to identify 15 homologs in *M. lacunae* (29–60% sequence identity) and 7 homologs in *M. stamsii* (28–66% sequence identity) (Figure 6).

The homologs with highest sequence identity to MspA for both *M. lacunae* (WP_109968644.1) and *M. stamsii* (WP_109940855.1) were investigated in more detail. Both proteins contain two Cys residues in the middle part of the protein, very similar to the MspA of *M. thermophila* (Dueholm et al., 2015) and might be able to form intermolecular disulfide bonds between different protein monomers thus stabilizing the sheath structure. Analysis with the AmylPred2 tool analysis showed similar regularly spaced amyloidogenic regions, especially for the *M. stamsii* protein WP_109940855.1 (Supplementary Figure S6). This protein also has a 1–23 signal peptide as seen for MspA. Finally, two homologs (WP_0426896011.1 and WP_007315077.1) were identified in the closely related species *Methanolinea tarda* (both 31% sequence identity) (data not shown). No homologs to the *M. termophila* MspA could be identified within the two available *Methanospirillum* genomes.

## Discussion

Microorganisms belonging to the methanogenic genera *Methanosaeta* and *Methanospirillum* can either occur as single cells (Southam et al., 1990) or as multicellular filaments enclosed within sheath structures where individual cells are separated by spacer plugs (Shaw et al., 1985; Firtel et al., 1993). The major sheath protein MspA has recently been identified in *M. thermophila* PT and represents the first example of a functional amyloid protein in Archaea (Dueholm et al., 2015). In this paper we report the identification of a novel MspA protein from the evolutionary distant species *M. hungatei* JF-1. The intact *M. hungatei* JF-1 archaea binds the amyloid-specific antibody WO1 and the purified sheaths reveal an extended β-sheet structure when investigated with FTIR, supporting the amyloid structure of the sheaths. The identified MspA protein is smaller than the previously identified MspA of *M. thermophila* PT (40.6 kDa compared to 60.9 kDa). This may explain why sheaths isolated from *Methanosaeta* (previously known as *Methanothrix*) are 0.3 μm wider than sheaths isolated from *M. hungatei* (Patel et al., 1986). We were not able to identify any other proteins, and therefore the proteins making up the spacer plugs remain unclear. These plug proteins are likely less abundant than the sheath proteins, but it cannot be ruled out that they are one of the PKD proteins or the hypothetical protein WP_011448920.1 listed in Supplementary Table S1. In this context, it is worth noting that the PDK proteins, so called because they have a domain homologous to one of the repeats found in the human polycystic kidney disease 1 protein, are connected to human functional amyloid: the protein Pmel17. This protein is proteolytically processed to form amyloid structures which incorporate melanin to form melanosomes within melanocytes. One of the two key domains involved in this process is a PKD domain (Watt et al., 2009).

Bioinformatic analysis of the *M. hungatei* JF-1 MspA protein revealed that the protein is overall amyloidogenic and has regularly spaced amyloidogenic regions. A 23 aa long signal peptide could be identified using the tool SignalP 5.0 tool. Possession of a signal peptide is a common feature for functional protein systems like the curli system in *E. coli* and the Fap system in many *Pseudomonas* strains (Taglialegna et al., 2016). Both of these systems rely on the transportation of the primary amyloid-forming protein – CsgA and FapC, respectively – across the inner membrane through the common Sec translocon and across the outer membrane through more specific membrane proteins (Cao et al., 2014; Rouse et al., 2017). The single proteins are assembled into amyloid fibrils after they enter the extracellular environment.

We have attributed the great stability and stiffness of the MspA-composed sheaths to a combination of functional amyloid structure and disulfide bonds connecting the individual hoops as originally proposed by Dueholm et al. (2015). Despite the fact that the *M. hungatei* JF-1 MspA only has a single Cys residue at position 271 it could still form intermolecular disulfide bonds with other MspA proteins and thereby stabilize the sheath. Recombinant expression of *M. thermophila* PT MspA showed that the protein readily fibrillates into amyloid structures, however it did not spontaneously form sheaths. Therefore, it was suggested that some accessory factors are required for sheaths to form. In the case of *M. hungatei*, these factors could be phenol-soluble polypeptides, which account for ≈20% of the sheaths (Southam and Beveridge, 1992). Removal of these polypeptides from the sheaths resulted in less rigid sheaths that have lost their original cylindrical shape. Recombining the polypeptide-free sheaths with the phenol-soluble polypeptide fraction could to some degree restore the rigidity of the sheaths (Southam and Beveridge, 1992), supporting the possibility that they might serve a scaffolding purpose.

In addition to the six internal homologs, 24 homologs to the novel *M. hungatei* JF-1 MspA were identified in related *Methanospirillum* and *Methanolinea* strains. The regularly spaced amyloidogenic regions in the proteins might be involved in formation of the cross-β-sheet structures that were observed with FTIR. As no homologs can be identified for the MspA from *M. thermophila* PT, we suggest that *M. hungatei* JF-1 MspA is a more common sheath protein. None of these MspA homolog proteins have any function assigned to them (they all appear as “hypothetical proteins” in the RefSeq database) and it therefore needs to be investigated in more detail if these proteins, when expressed recombinantly, can in fact form functional amyloids. Also, as mentioned, even a low sequence similarity between amyloid proteins would not necessarily compromise their ability to form amyloids. We would not be surprised if the number of homologs increases as the number of sequenced archaeal genomes increases.

## Author Contributions

MD, PN, and DO designed the study. KF and LH cultured the archaea. LC, MD, and KF purified the cell wall sheaths. MD performed antibody staining and confocal laser scanning microscopy. GC performed transmission electron microscopy. LC performed Fourier-transform infrared spectroscopy. MD did the depolymerization and performed the MS/MS analysis of the sheaths. LC and MD did the bioinformatic analyses of MspA. MD did the genome sequencing of *Methanospirillum* type strains. LC, MD, and DO wrote the paper. All authors reviewed the results and approved the final version of the manuscript.

## Conflict of Interest Statement

The authors declare that the research was conducted in the absence of any commercial or financial relationships that could be construed as a potential conflict of interest.

## Footnotes

1 http://www.cbs.dtu.dk/services/SignalP/

## Abbreviations

aa: amino acids
DTT: dithiothreitol
FTIR: Fourier-transform infrared
RefSeq: NCBI Reference Sequence database
TEM: transmission electron microscopy
