# Supplementary material for: The Sheaths of Methanospirillum Are Made of a New Type of Amyloid Protein
Source: Front Microbiol. 2018 Nov 13;9:2729. doi: 10.3389/fmicb.2018.02729 (PMC6242892; doi:10.3389/fmicb.2018.02729)
Supplement: Supplementary file 1 [file Data_Sheet_1.docx]

The sheaths of *Methanospirillum* are made of a new type of amyloid protein

Line Friis Bakmann Christensen, Lonnie Maria Hansen, Kai Finster, Gunna Christiansen, Per Halkjær Nielsen, Daniel Otzen and Morten Simonsen Dueholm

Supplementary Material


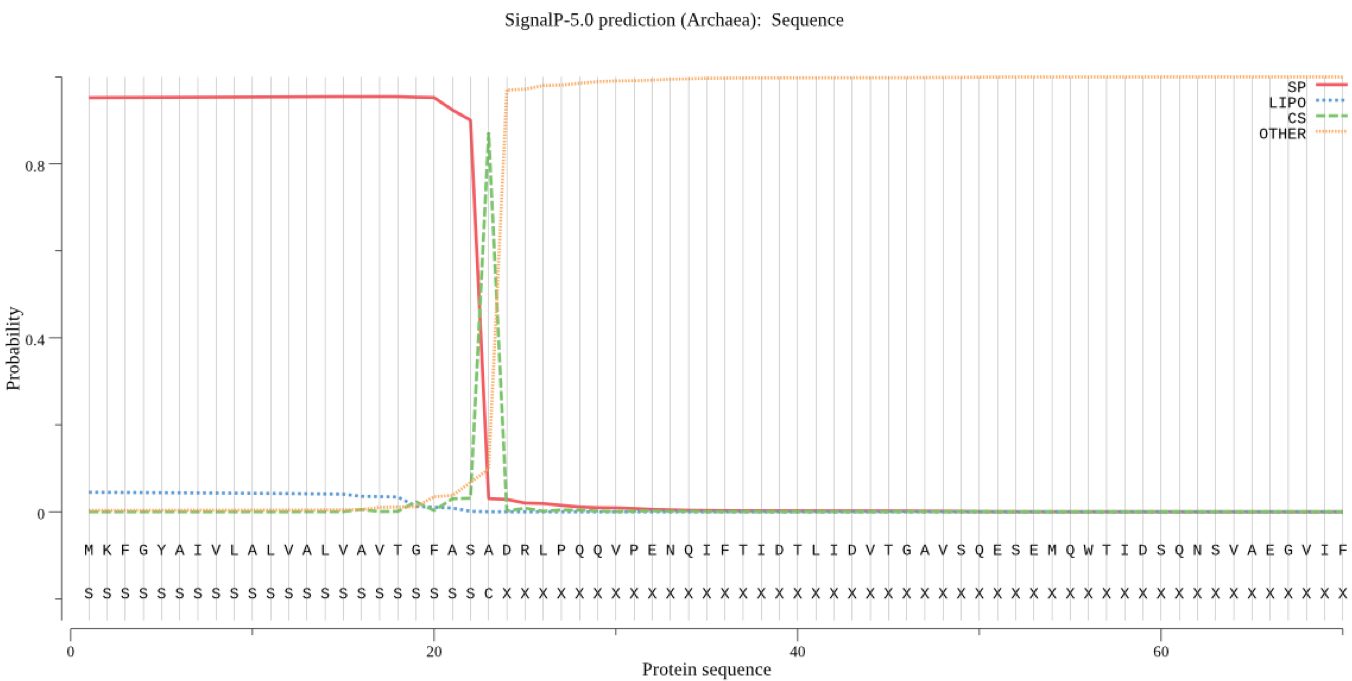


**Figure S1.** ***M. hungatei* JF-1 MspA contains a signal peptide.** SignalP 5.0 (not yet available as online tool) was used to confirm the presence of an N-terminal signal peptide between residues 1 and 23 in MspA.


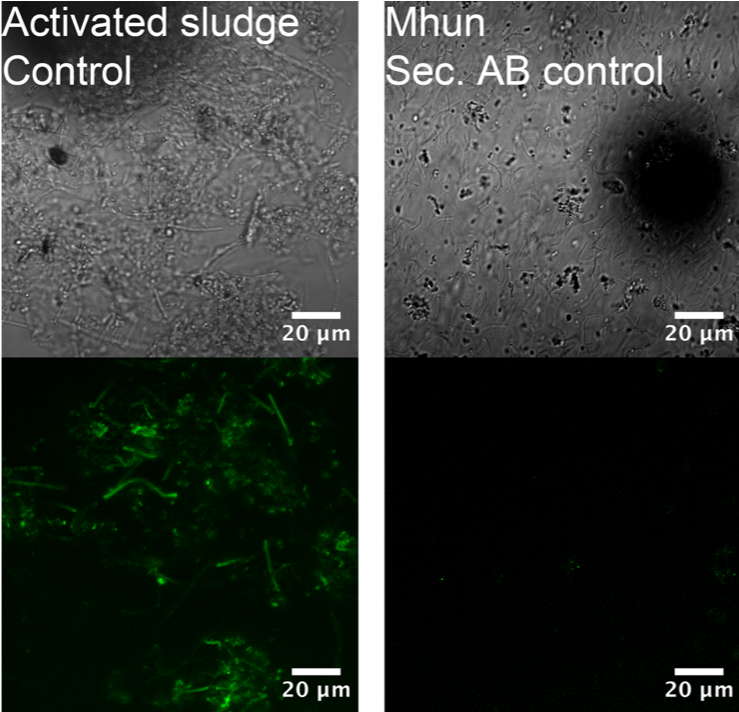


**Figure S2. *Methanospirillum* sheaths bind the amyloid-specific conformational antibody WO1 without showing unspecific binding to the secondary antibody**. Samples with only the secondary antibody and either an activated sludge control sample or intact *Methanospirillum hungatei* GP1 (Mhun) filaments were analyzed to rule out unspecific binding of the secondary antibody. **Top**: a differential interference contrast image. **Bottom**: green fluorescence signal from the antibody. Scale bars are shown in each picture.

**
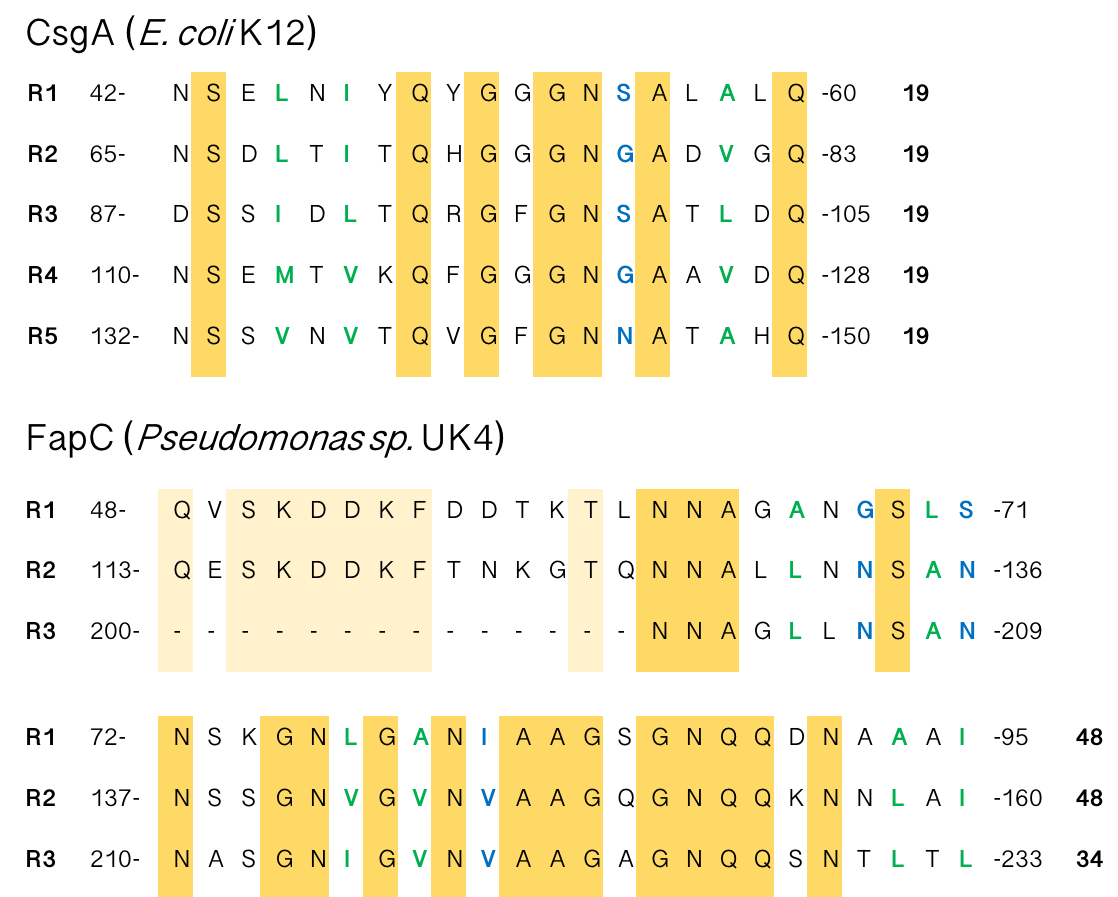
**

**Figure S3. Repeats in CsgA and FapC have many conserved Gln (Q), Asn (N), Gly (G), Ala (A) and Ser (S) residues.** CsgA (top) has five imperfect repeats (R1-R5) while FapC (bottom) has three (R1-R3). Conserved residues are colored yellow. Residues with similar properties are shown in green (small and/or hydrophobic) or blue (polar + Gly). For FapC the residues colored light yellow are only conserved between R1 and R2.

**
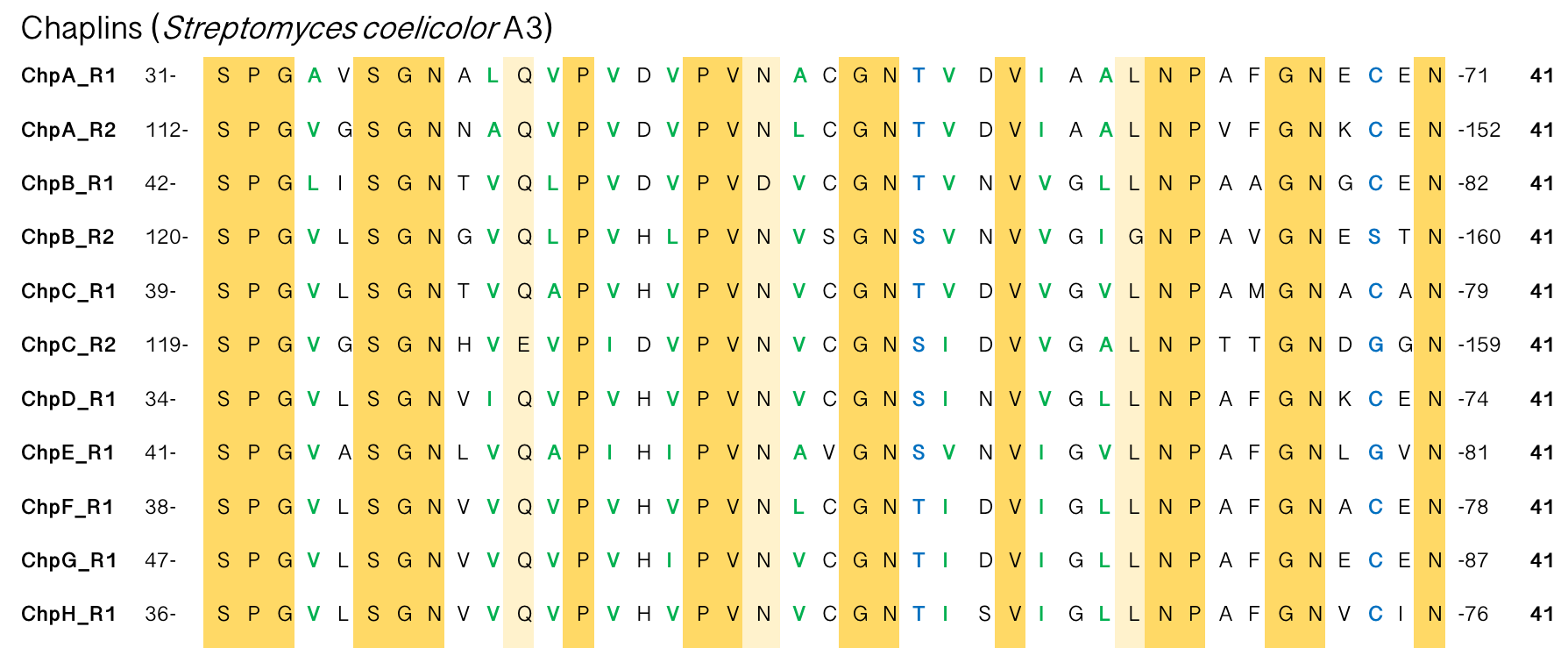
**

**Figure S4.** **Repeats in chaplin proteins have many conserved Asn (N), Gly (G), Pro (P), Val (V) and Ser (S) residues.** The chaplin proteins from *Streptomyces coelicolor* A3 have 1 (ChpD-H) or 2 (ChpA-C) imperfect repeats. Conserved residues are colored yellow. Residues with similar properties are shown in green (small and/or hydrophobic) or blue (polar + Gly). Residues where only one repeat is different from the rest is colored light yellow.

**
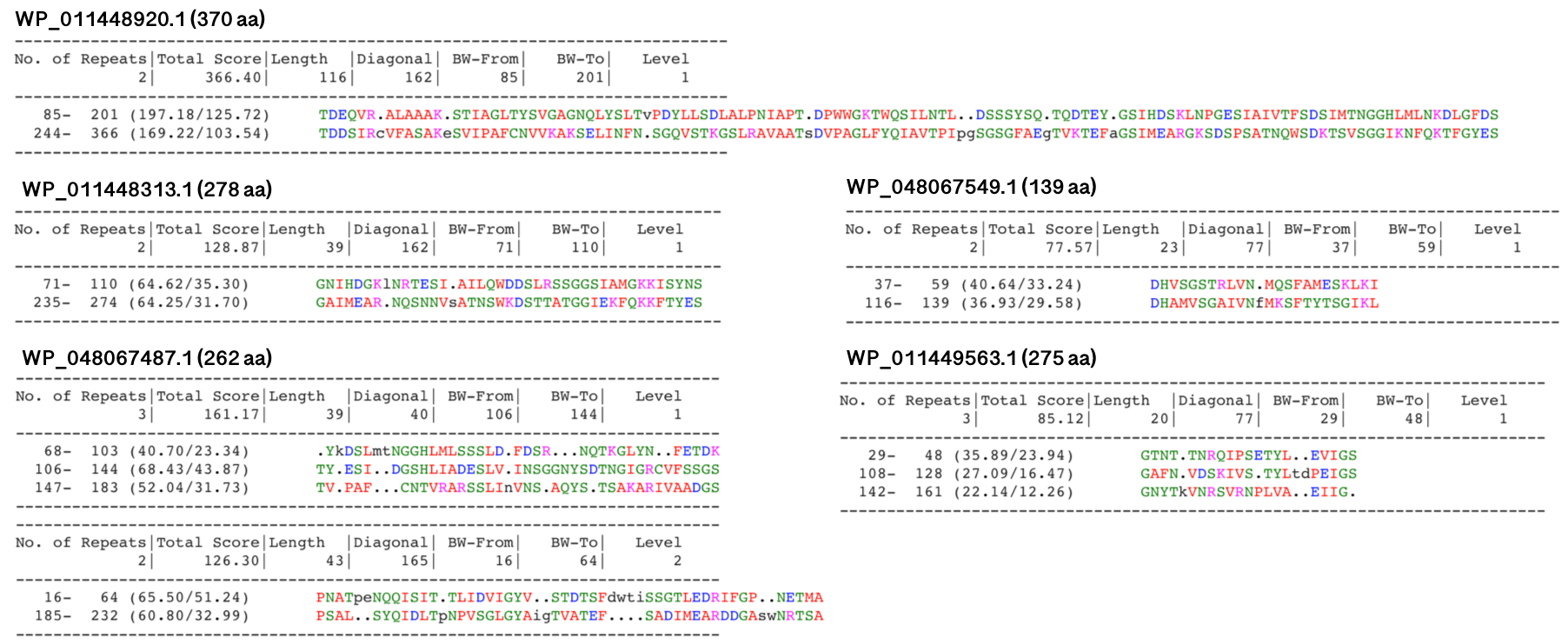
**

**Figure S5.** **MspA internal homologs have imperfect repeats of varying length.** Analysis with RADAR (Andreas and Liisa, 2000) showed that five of the internal homologs contained imperfect repeats of different lengths.


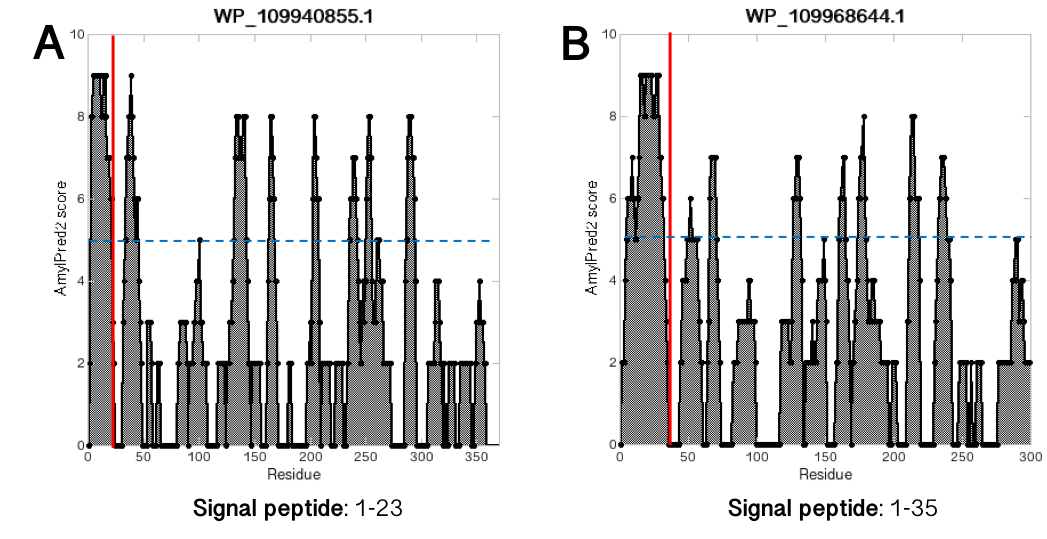


**Figure S6.** **MspA homologs in closely related *Methanospirillum* strains share many of the same sequence features as MspA.** (A) WP_109940855.1 from *Methanospirillum stamsii* and (B) WP_109968644.1 from *Methanospirillum lacunae* were analyzed with AmylPred2 (Tsolis et al., 2013) and shown to have more or less regularly spaced regions of proposed high amyloidogenicity.

**Table S1. Protein identified by MS/MS after disassembly of sheaths from *Methanospirillum hungatei* GP1.** The sheaths were disassembled using DTT and NaOH and run on SDS-PAGE after which all protein bands were analysed with MS/MS. The combined data for all protein bands are shown. *Good correlation between protein molecular weight and gel migration. **The Exponentially Modified Protein Abundance Index (EmPAI) offers approximate, label-free, relative quantifications of the proteins in a mixture based on protein coverage by the peptide matches in a database search result (Ishihama et al., 2005). The protein shown in bold blue is MspA.

| Uniprot accession number | RefSeq accession number | RefSeq protein description | Molecular weight (kDa)* | Mascot score | Protein sequence coverage (%) | Spectral MS/MS counts (Mascot) | EmPAI (Mascot) ** |
| --- | --- | --- | --- | --- | --- | --- | --- |
| **Q2FRN9_METHJ** | **WP_011449234.1** | **Hypothetical protein** | **40.6** | **43255** | **74** | **1407** | **38.57** |
| Q2FPL5_METHJ | WP_011447477.1 | DUF3821 domain-containing protein | 142.5 | 1455 | 18 | 82 | 0.65 |
| Q2FPM0_METHJ | WP_011447478.1 | DUF3821 domain-containing protein | 136.3 | 1320 | 20 | 97 | 0.81 |
| Q2FPL7_METHJ | WP_011447475.1 | PKD domain-containing protein | 106.2 | 1103 | 14 | 65 | 0.58 |
| Q2FRU4_METHJ | WP_011449401.1 | PKD domain-containing protein | 102.1 | 1038 | 19 | 57 | 0.51 |
| Q2FPL6_METHJ | WP_011447476.1 | DUF3821 domain-containing protein | 121.7 | 596 | 11 | 45 | 0.34 |
| Q2FMC0_METHJ | WP_011448920.1 | Hypothetical protein | 39.4 | 435 | 32 | 26 | 0.62 |
| Q2FLP4_METHJ | WP_011447640.1 | PKD domain-containing protein | 200.3 | 276 | 4 | 20 | 0.08 |
| Q2FTS9_METHJ | WP_011449220.1 | 5,10-methylenetetrahydromethanopterin reductase | 35.1 | 105 | 16 | 12 | 0.57 |
| Q2FSN1_METHJ | WP_011449111.1 | Coenzyme-B sulfoethylthiotransferase subunit gamma | 28.7 | 87 | 13 | 3 | 0.39 |
| Q2FKZ0_METHJ | WP_011448823.1 | Hydrogenase iron-sulfur subunit | 15.9 | 86 | 24 | 5 | 1.16 |

**References**

Andreas, H., and Liisa, H. (2000). Rapid automatic detection and alignment of repeats in protein sequences. *Proteins: Structure, Function, and Bioinformatics* 41**,** 224-237.

Ishihama, Y., Oda, Y., Tabata, T., Sato, T., Nagasu, T., Rappsilber, J., and Mann, M. (2005). Exponentially modified protein abundance index (emPAI) for estimation of absolute protein amount in proteomics by the number of sequenced peptides per protein. *Mol Cell Proteomics* 4**,** 1265-1272.

Tsolis, A.C., Papandreou, N.C., Iconomidou, V.A., and Hamodrakas, S.J. (2013). A consensus method for the prediction of 'aggregation-prone' peptides in globular proteins. *PLoS One* 8**,** e54175.
